# Draft Genome Sequences of Colistin-Resistant MCR-1-Producing *Escherichia coli* ST1850 and ST74 Strains Isolated from Commercial Chicken Meat

**DOI:** 10.1128/genomeA.00329-17

**Published:** 2017-05-18

**Authors:** Daniel F. Monte, Miriam R. Fernandes, Louise Cerdeira, Tiago A. de Souza, Andressa Mem, Bernadette D. G. M. Franco, Mariza Landgraf, Nilton Lincopan

**Affiliations:** a Department of Food and Experimental Nutrition, School of Pharmacy, University of São Paulo, São Paulo, Brazil; b Food Research Center, University of São Paulo, São Paulo, Brazil; c Department of Clinical Analysis, School of Pharmacy, University of São Paulo, São Paulo, Brazil; d Genome Investigation and Analysis Laboratory (GENIAL), Institute of Biomedical Sciences, University of São Paulo, São Paulo, Brazil; e Department of Veterinary Medicine, Federal University of Paraná, Paraná, Brazil; f Department of Microbiology, Institute of Biomedical Sciences, University of São Paulo, São Paulo, Brazil

## Abstract

We present here the draft genome sequences of two colistin-resistant *mcr-1*-carrying *Escherichia coli* strains belonging to sequence type 74 (ST74) and ST1850, isolated from commercial chicken meat in Brazil. Assembly of this draft genome resulted in 5,022,083 and 4,950,681 bp, respectively, revealing the presence of the IncX4 plasmid-mediated *mcr-1* gene responsible for resistance to colistin.

## GENOME ANNOUNCEMENT

Colistin-resistant *Escherichia coli* strains carrying the *mcr-1* gene have been widely identified in livestock (1), where the poultry production chain could contribute to the silent dissemination of this gene (2). In this regard, the use of colistin (as a growth promoter) in food-producing animals has been pointed out as an important factor contributing to the emergence, persistance, and dissemination of the *mcr-1* gene (3, 4). Recently, we have reported the identification of MCR-1-positive *E. coli* strains in commercial chicken meat in South America (3). We hereby present the draft genome sequences of two colistin-resistant *mcr-1*-carrying *E. coli* strains belonging to sequence type 74 (ST74) and ST1850, isolated in 2016 in Brazil.

*E. coli* strains CF111 and CF341 were isolated using traditional methods, according to the FDA (5). Genomic DNA of these isolates was extracted and sequenced using the MiSeq version 3 platform paired-end reads (300 × 300 bp) (Illumina, San Diego, CA). *De novo* assembly was performed using SPAdes version 3.9.0 (6). This assembly was curated using Geneious version R9 (Biomatters Ltd., New Zealand) and submitted for annotation using NCBI Prokaryotic Genome Annotation Pipeline version 3.2. Multilocus sequence types (MLST), plasmid replicons, antimicrobial resistance genes, and *E. coli* virulence genes were identified using multiple databases: MLST 1.8, PlasmidFinder 1.3, ResFinder 2.1, and VirulenceFinder 1.5, respectively (http://genomicepidemiology.org/).

*E. coli* CF111 and CF341 belonged to ST1850 and ST74, presenting 137 and 96 contigs distributed in genomes of 4,950,681 bp and 5,022,083 bp in size, respectively. In brief, CF111 presented 5,177 protein-coding genes, 55 RNA-coding genes (46 tRNAs, 2 rRNAs, and 7 noncoding RNAs [ncRNAs]), and 314 pseudogenes, with a G+C content of 50.7%, whereas CF341 presented 5,284 protein-coding genes, 64 RNA-coding genes (50 tRNAs, 1 rRNAs, and 13 noncoding RNAs [ncRNAs]), and 356 pseudogenes, with a G+C content of 50.6%. *In silico* detection of plasmids identified IncX4, IncFIB, and IncI1 in both isolates. On the other hand, IncFIC and IncFIA were identified in *E. coli* CF111, whereas IncFII and IncFIB were identified in *E. coli* CF341. In this regard, IncX4-type plasmids have been key vectors responsible for the dissemination of the *mcr-1* gene in *E. coli* strains in food, humans, and animals in Brazil (3, 7, 8).

In addition to the *mcr-1* gene, while *E. coli* CF111 carried the β-lactam resistance gene *bla*_CMY-2_ and aminoglycoside resistance genes *aadA12* and *aph(3′)-Ic*, *E. coli* CF341 harbored the β-lactam resistance gene *bla*_CTX-M-2_, aminoglycoside resistance genes *aadA1* and* aadA2*, and sulfonamide resistance genes *sul1, sul2,* and *sul3*. Moreover, VirulenceFinder 1.5 identified i*ss, ipfA,* and *gad* in *E. coli* CF341 and *iroN, gad, tsh, iss,* and *mchF* virulence genes in *E. coli* CF111.

In summary, we report the draft genome sequences of two colistin-resistant *mcr-1*-carrying *E. coli* strains belonging to ST74 and ST1850, isolated in 2016 from commercial chicken meat in Brazil. Whole-genome sequence (WGS) analysis indicates that these strains carried the *mcr-1* gene on IncX4-type plasmids, as previously reported in food, human, and animal *E. coli* strains from Brazil (3, 7, 8). These draft genome sequences could contribute to providing data to better understand the molecular mechanisms leading to the dissemination and successful flow of *mcr-1*-harboring *E. coli* strains in human, animal, and food production.

### Accession number(s).

The genome sequences of *E. coli* strains CF111 and CF341 have been deposited at DDBJ/ENA/GenBank with accession numbers MUIP00000000 and MUIQ00000000, respectively.
